# Transcriptional regulation of human sperm-associated antigen 16 gene by S-SOX5

**DOI:** 10.1186/s12867-017-0082-3

**Published:** 2017-01-31

**Authors:** Ling Zhang, Yunhao Liu, Wei Li, Qiaoling Zhang, Yanwei Li, Junpin Liu, Jie Min, Chaofan Shuang, Shizheng Song, Zhibing Zhang

**Affiliations:** 1 School of Public Health, Wuhan University of Science and Technology, Wuhan, 430065 Hubei China; 2 Department of Obstetrics and Gynecology, Virginia Commonwealth University, Richmond, VA 23298 USA; 3 Central China Normal University, Wuhan, Hubei 430000 China; 4 Department of Computer Science, Wellesley College, Wellesley, MA 02481-5701 USA; 5 Wuhan Hospital for the Prevention and Treatment of Occupational Diseases, Wuhan, 430000 Hubei China

**Keywords:** S-SOX5, SPAG16L, Transcriptional regulation, Central apparatus, Cilia

## Abstract

**Background:**

The mammalian sperm-associated antigen 16 gene (*Spag16*) uses alternative promoters to produce two major transcript isoforms (*Spag16L* and *Spag16S*) and encode proteins that are involved in the cilia/flagella formation and motility. *In silico* analysis of both mouse and human *SPAG16L* promoters reveals the existence of multiple putative SOX5 binding sites. Given that the *SOX5* gene encodes a 48-kDa transcription factor (S-SOX5) and the presence of putative SOX5 binding sites at the *SPAG16L* promoter, regulation of *SPAG16L* expression by S-SOX5 was studied in the present work.

**Results:**

S-SOX5 activated human *SPAG16L* promoter activity in the human bronchial epithelia cell line BEAS-2B cells. Mutation of S-SOX5 binding sites abolished the stimulatory effect. Overexpression of S-SOX5 resulted in a significant increase in the abundance of *SPAG16L* transcripts whereas silencing of S-SOX5 by RNAi largely reduced the *SPAG16L* expression. Chromatin immunoprecipitation assays showed that S-SOX5 directly interacts with the *SPAG16L* promoter.

**Conclusion:**

S-SOX5 regulates transcription of human *SPAG16L* gene via directly binding to the promoter of *SPAG16L*. It has been reported that expression of sperm-associated antigen 6 (*SPAG6*), encoding another axonemal protein, is activated by S-SOX5. Therefore, S-SOX5 may regulate formation of motile cilia/flagella through globally mediating expression of genes encoding axonemal proteins.

## Background

The family of Sox transcription factors is defined by the presence of a conserved high mobility-group (HMG) domain that mediates DNA-binding and is highly similar to that of the sex-determining region (SRY) protein [1, 2]. Based on phylogenic analysis of HMG domain sequences and full-length protein sequences/functional features, *Sox* genes are classified into 10 groups from A to J [3]. They display distinct tissue-specific expression patterns and have been implicated in regulation of a wide range of developmental processes [1]. SOX proteins exert gene activation or repression by binding to a consensus DNA motif, with or without aid of other transcription factors [1, 4]. Available evidence indicates that a particular SOX protein can mediate expression of various target genes through recognizing different binding sites during the formation of many tissues [1, 5]. Selection of specific target genes by SOX proteins depends on flanking sequences of the consensus core, homo-/hetero-dimerization of SOX proteins at recognition sites and association with other transcription factors [1, 6].

The SOXD group is composed of SOX5, SOX6 and SOX13 [1]. Human *SOX5* is primarily expressed in the short (*S*-*SOX5*) and long form (*L*-*SOX5*) of transcripts [7, 8]. *L*-*SOX5* cDNA is predicted to encode a 763-amino-acid protein that exceeds S-SOX5 by 416 residues [8]. S-SOX5, which lacks N-terminal domain required for dimerization with other SOXD proteins, is predominantly detected in testis and brain while L-SOX5 is expressed in multiple tissues including testis, heart, liver and skeletal muscle [7, 8]. The difference in the protein structure and tissue distribution between the two forms of SOX5 implies distinct biological functions for these isoforms. The two SOX5 isoforms are conserved in mouse [9]. Mouse *S*-*Sox5* was originally cloned from testis [10]. The restricted presence of mouse orthologue S-SOX5 proteins in round spermatids and regulation of testis-related gene expression by S-SOX5 suggests that S-SOX5 plays a specialized role in spermatogenesis within the testis [10–13]. Later studies demonstrated that mouse S-SOX5 is also expressed in the lung and brain, tissues bearing motile cilia [9], and it is capable of activating expression of sperm-associated antigen 6 gene (*Spag6*), whose translated product is enriched in the tissues with motile cilia, particularly in the testis [9].

Mammalian sperm-associated antigen 16 (*Spag16*) is the orthologue of *Chlamydomonas reinhardtii pf20* that encodes an axonemal protein essential for flagellar motility. *Chlamydomonas* mutants carrying *pf20* mutation display paralyzed flagella with defects in axonemal central apparatus [14]. Both mouse and human *SPAG16* genes are expressed as two major transcripts of 1.4 and 2.5 kb with different expression patterns. The human 1.4 kb transcript was detected in multiple tissues whereas the human 2.5 transcript was highly expressed in testis [15, 16]. Mouse 2.5 kb transcript has a similar tissue distribution as the human orthologue; however, the 1.4 kb transcript is only present in mouse testis [17]. The translated 71 kDa (SPAG16L) and 35 kDa (SPAG16S) proteins have different locations and functions in male germ cells. SPAG16L is located in the axoneme central apparatus of sperms and plays a crucial role in sperm motility. Besides the similar localization as SPAG16L, SPAG16S is also present in the nucleus of post-meiotic germ cells and seems to be essential for viability of these cells during spermatogenesis [16, 18, 19].

Given that both S-SOX5 and SPAG16L are present in tissues containing cells with motile cilia/flagella, it is hypothesized that expression of *SPAG16L* is regulated by S-SOX5. In the present work, we report bioinformatic and biochemical characterization of the human *SPAG16L* promoter. The in silico prediction showed multiple putative binding sites for SOX5 in the *SPAG16L* promote region. The empirical evidence revealed that S-SOX5 activates expression of *SPAG16L* via direct interaction with SOX5 binding sites at the *SPAG16L* promoter.

## Results

### S-SOX5 stimulates human *SPAG16L* promoter in BEAS-2B cells

**Fig. 1:**
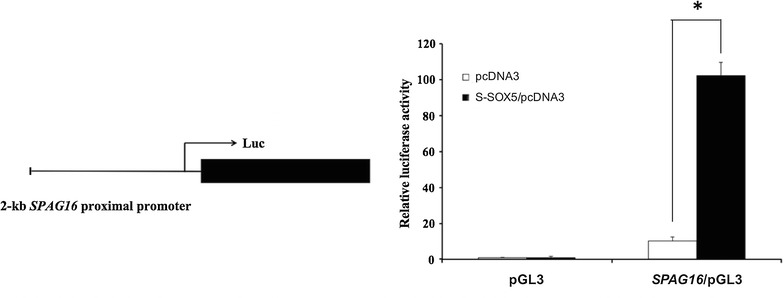
Effect of S-SOX5 on the human *SPAG16L* promoter activity. 2-kb human *SPAG16L* promoter construct was transfected to BEAS-2B cells. The cells were also co-transfected with S-SOX5/pcDNA3 or empty pcDNA3 plasmid. The promoter activity was measured by dual luciferase assays after 48 h transfection. Values indicate mean ± S.E. (*error bars*) of the relative luciferase activity (n = 3). *p < 0.05 (Student’s *t* test) compared to the control

We have previously used the ConSite program to predict transcription factors of human *SPAG16L* and found multiple putative SOX5-binding sites in the 2-kb proximal promoter region of *SPAG16L* [9]. To explore the influence of S-SOX5 on expression of *SPAG16L*, a transcriptional *luc* fusion was made by cloning a 2-kb DNA fragment containing the *SPAG16L* proximal promoter into pGL3-basic vector. The resultant construct (SPAG16/pGL3) was co-transfected with S-SOX5 expression plasmids (S-SOX5/pcDNA3) into human bronchial epithelial cells BEAS-2B and the relative luciferase activity was measured. Endogenous expression of *S*-*SOX5* has been reported in BEAS-2B cells, suggesting this cell line can be used to study S-SOX5-mediated gene regulation [9]. The basal level of *SPAG16L* expression was detected, which is about 10× higher than that of the control empty pGL3-basic plasmids. When co-transfected with S-SOX5, the relative luciferase activity was elevated about 100-folds (Fig. 1). These results indicate that S-SOX5 stimulates transcription of human *SPAG16L*.

### Levels of *SPAG16L* mRNA are elevated by exogenous S-SOX5

**Fig. 2:**
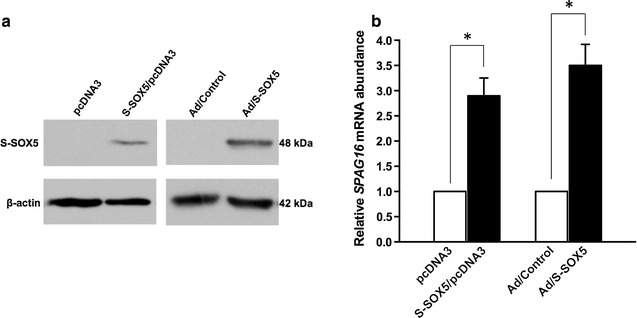
Exogenous S-SOX5 up-regulates *SPAG16L* mRNA expression in BEAS-2B cells. **a** Western blot analysis of S-SOX5 protein levels in BESA-2B cells stably expressing S-SOX5 (*left*) or infected with AdS-SOX5 (*right*) detected with the Pico system. **b** Analysis of *SPAG16L* mRNA expression by real-time PCR. *GAPDH* was also amplified simultaneously to normalize the results. Values indicate mean ± S.E. (*error bars*) of the relative mRNA level (n = 3). *p < 0.05 (Student’s *t* test) compared to the controls

The effect of S-SOX5 on the expression level of *SPAG16L* mRNA in BEAS-2B cells was examined. To this end, BEAS-2B cells stably expressing S-SOX5 (S-SOX5/pcDNA3) or infected with S-SOX5 adenovirus (Ad/S-SOX5) were generated, and expression of S-SOX5 protein in these cells was confirmed by Western blot analysis. Even though, S-SOX5 was undetected in the controls, the S-SOX5 protein (48 kDa) was produced in both cell lines and clearly increased when infected with S-SOX5 adenovirus (Fig. 2a). To measure the relative abundance of *SPAG16L* mRNA in the same cells, real-time PCR was performed. As expected, the relative level of *SPAG16L* mRNA in both cell types was significantly higher than that in the control (Fig. 2b). The results show that exogenous S-SOX5 is able to increase *SPAG16L* mRNA levels.

### Knockdown of S-SOX5 in BEAS-2B cells reduces expression of *SPAG16L*

**Fig. 3:**
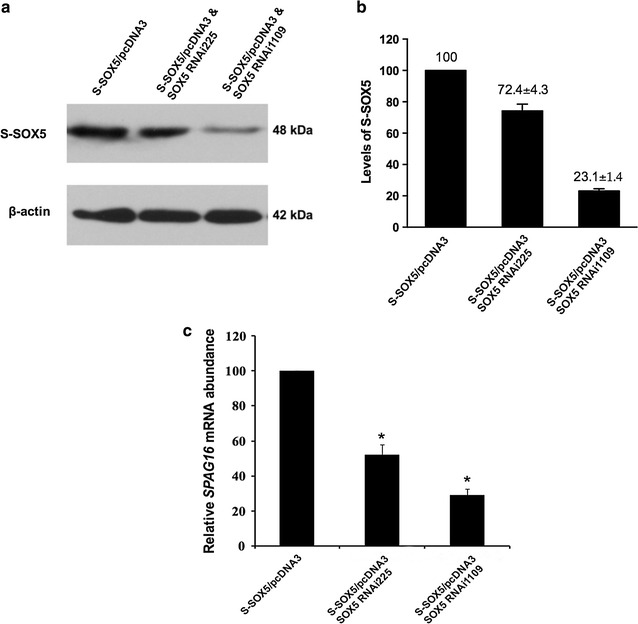
Knockdown of S-SOX5 in BEAS-2B cells by RNAi results in decrease of *SPAG16L* mRNA. **a** Western blot analysis of S-SOX5 proteins in BEAS-2B cells stably expressing two RNAi constructs. **b** After RNAi treatment, the levels of S-SOX5 in BEAS-2B cells were estimated by analysing intensity of protein bands using ImageJ software. **c** Analysis of *SPAG16L* mRNA expression by real-time PCR in BEAS-2B cells transfected with two SOX5 RNAi constructs. Silencing of S-SOX5 by RNAi reduced expression of *SPAG16L.* Values indicate mean ± S.E. (*error bars*) of the relative mRNA level (n = 3). *p < 0.05 (Student’s *t* test) compared to the control

To further investigate the regulatory role of S-SOX5 on *SPAG16L* expression, two human *SOX5* RNAi constructs that respectively target portion 225-246 and 1109-1130 of the *SOX5* transcript were used [9]. The BEAS-2B cells were co-transfected with these constructs or SOX5 expression plasmids (S-SOX5/pcDNA3). The efficiency of the *SOX5* RNAi plasmids was examined by Western blot analysis. Both RNAi constructs were able to decrease S-SOX5 protein levels, but the construct targeting portion 1109-1130 of the *SOX5* transcript led to a remarkable reduction of S-SOX5 (Fig. 3a, b). Moreover, the abundance of *SPAG16L* mRNA in the BEAS-2B cells was measured by real-time PCR. It was decreased correspondingly with two different RNAi constructs (Fig. 3c). These data suggest that expression of *SPAG16L* depends on the activity of S-SOX5 protein.

### S-SOX5 binds to the human *SPAG16L* promoter

**Fig. 4:**
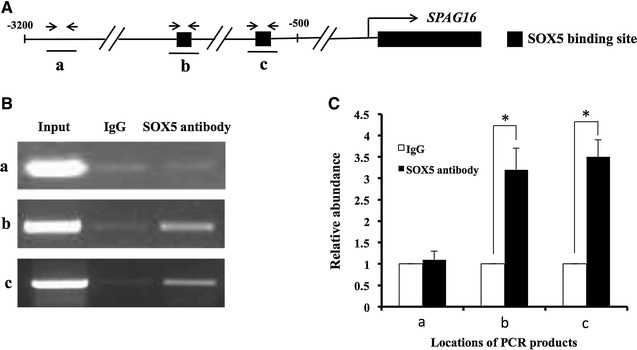
S-SOX5 associates with the human *SPAG16L* promoter as revealed by ChIP assay. **A** Schematic representation of the human *SPAG16L* proximal promoter and the regions (*a*–*c*) amplified by ChIP primers used in this study. *Arrows* show the location of the primers. **B** Representative ChIP assay results with BEAS-2B cells infected by AdS-SOX5 using a control rabbit IgG or an antibody specifically against SOX5. *a* with a primer set not flanking any potential SOX5 binding sites; *b* and *c*, with primer sets flanking potential SOX5 binding sites. **C** qPCR quantification of ChIP products. DNA recovered from ChIP was used as a template for real-time PCR analysis. Data shown are mean ± S.E. (*error bars*) of three independent replicates. *p < 0.05 (Student’s *t* test) compared to the normal rabbit IgG pulldown group

To study the molecular interaction between S-SOX5 and the *SPAG16L* promoter, BEAS-2B cells were infected with S-SOX5 adenovirus (AdS-SOX5) and ChIP assay was conducted with an antibody specifically against S-SOX5 or rabbit IgG. Two primer sets flanking putative SOX5 binding sites were designed; as a control, another primer set located about 3 kb upstream of the transcriptional start site without flanking any SOX5 binding site was also designed (Fig. 4A). Compared to using the rabbit IgG, more PCR products were amplified by two PCR primer sets flanking putative SOX5 binding sites when ChIP was performed using the SOX5 antibody (Fig. 4B, b, c). However, the control primer set was unable to amplify more specific DNA fragments (Fig. 4B, a). The result was consistent with the statistical analysis of the relative abundance of the PCR products (Fig. 4C). These results suggest that S-SOX5 is capable of binding to the promoter of *SPAG16L.*

### Mutation of SOX5 binding sites abolishes activation of the *SPAG16L* promoter in BEAS-2B cells

**Fig. 5:**
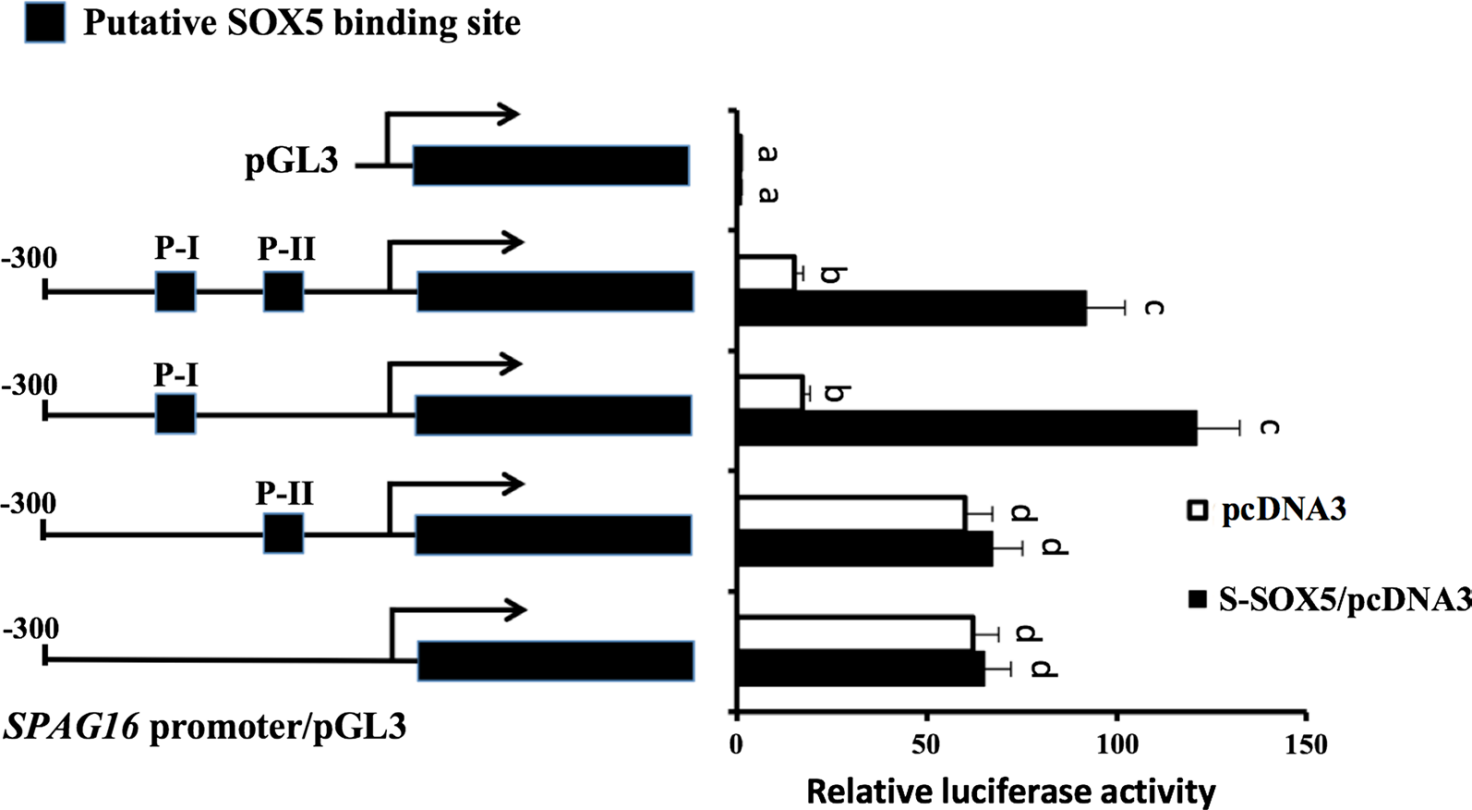
Functional analyses of S-SOX5 binding sites in the human *SPAG16L* promoter. *Left* maps of the wild-type human *SPAG16L* promoter construct containing two putative SOX5 binding sites (P-I & P-II) and the constructs with mutations of the potential SOX5 binding sites.* Right*, effect of S-SOX5 on the function of the wild-type and mutated *SPAG16L* promoter. BEAS-2B cells were co-transfected with *SPAG16L* promoter constructs and either pcDNA3 control or S-SOX5/pcDNA3. Relative luciferase activity, normalized to pGL3 control plasmids co-transfected with pcDNA3 vectors. Values are mean ± S.E. (*error bars*) of the relative luciferase activity (n = 3). *Bars* labeled with *different letters* are significantly different (*p* < 0.05; two-way ANOVA with Tukey’s HSD test)

To study if S-SOX5 activates *SPAG16L* transcription via interacting with the SOX5 binding sites, a 300-bp *SPAG16L* promoter construct encompassing two putative SOX5 binding sites was generated. The resultant transcriptional fusion construct was transfected into BEAS-2B cells and relative luciferase activity was analyzed. The *SPAG16L* promoter showed basal level activity which is about 10× higher than that of the control (pGL3 only). As expected, co-transfection of S-SOX5 largely stimulated the *SPAG16L* promoter activity when both putative SOX5 binding sites (P-I & P-II) were present (Fig. 5). Mutation in the P-II site had little effect on the elevation of *SPAG16L* promoter activity, suggesting that the P-II site is not required for S-SOX5-mediated activation of *SPAG16L*. Interestingly, mutations in either the P-I site or both sites (P-I & P-II) led to higher baseline promoter activities; however, the promoter activities were not increased in the presence of S-SOX5 (Fig. 5). The results suggest that the P-I site probably contributes to the repression of *SPAG16L* by unknown factor(s) in the absence of S-SOX5 and is also essential for activation of *SPAG16L* by S-SOX5.

## Discussion

Cilia are evolutionarily conserved, filamentous cellular structures that are present on the cell surface and have been implicated in the sensing of environmental signals and cellular motility [20]. Based on the axonemal architectures, cilia can be classified into two major forms: “9 + 0” and “9 + 2” axonemal arrangements [20]. Primary cilia contain a “9 + 0” axoneme and usually non-motile, and detect mechanical and chemical signals from the surrounding environment. Dysfunction of primary cilia can lead to various human diseases including primary ciliary dyskinesia, polycystic kidney disease and retinal degeneration [21]. Motile cilia have a “9 + 2” axoneme that is composed of nine doublet microtubules and a central pair of microtubules. The associated structures of the “9 + 2” axoneme such as radial spokes and dynein arms are crucial for mediation of cilia motility [20]. Motile cilia are widely present in mammalian tissues including trachea, brain, spinal canal and sperm. Defects in motile cilia have been linked to diverse symptoms including hydrocephalus, sinusitis and bronchiectasis, situs inversus and male infertility [22].

Fine-tuned regulatory mechanisms mediate expression of stage-specific genes for the formation of distinct types of cilia during ciliogenesis. The sophisticated genetic program of ciliogenesis is modulated probably through the precise presence and maintenance of essential proteins in a time- and tissue-dependent manner [20, 23]. Empirical evidence shows that expression of ciliary genes is transcriptionally regulated and some transcriptional factors involved in ciliogenesis have been identified. These transcription factors include: HNF1β (hepatocyte nuclear factor 1β) [24], FKH-2 (forkhead 2) [25], RFX family of transcription factors [26], and FOXJ1 transcription factors [27]. Among them, RFX and FOXJ1 are two major transcriptional factors that control ciliogenesis. RFX proteins function as transcriptional regulators that interact with the X-box motif at MHC class II gene promoters [26, 28]. Functional analyses of RFX regulators indicate that they are required for modulating expression of key genes involved in different stages of ciliogenesis, including formation of ciliated sensory neurons, basal body migration and membrane docking, intraflagellar transport and ciliary motility [29–32].

FOXJ1 belongs to the forkhead/winged-helix family of transcriptional factors [23]. Loss-of-function analyses of *Foxj1* demonstrate the requirement of this gene for biosynthesis of motile cilia in mouse tissues [33, 34]. A number of target genes of FOXJ1 that are involved in ciliary motility have been identified in model organisms such as zebrafish and Xenopus. This suggests that FOXJ1 is a master transcriptional factor of motile ciliogenesis [35, 36].

Our earlier studies demonstrated that S-SOX5, together with FOXJ1, regulates expression of an axonemal gene, *Spag6* [9]. To explore if S-SOX5 also regulates other genes that are essential for motile cilia structure and/or function, and functions as a general transcription factor to control motile cilia/function, we decided to investigate if S-SOX5 regulates another axonemal central apparatus gene, *SPAG16L*, because the *SPAG16L* proximal promoter region also contains multiple putative SOX5 binding sites. Our findings demonstrated that S-SOX5 does regulate *SPAG16L* transcription through binding to the SOX5 binding sites. However, it should be aware that not all the putative SOX5 binding sites predicted by bioinformatic analysis are functional. One of the two putative SOX5 binding sites analysed in this study is not bound by S-SOX5. Thus, experiments must be conducted to verify if these putative binding sites are functional. Overall, this study presents another example that S-SOX5 regulates another gene essential for motile cilia function, and supports the notion that S-SOX5 is a general transcription factor to control formation and function of motile cilia.

Sperm flagella are special motile cilia. During spermiogenesis, germ cells undergo dramatic morphological changes as they develop into functional sperm. These changes include formation of flagella. Sperm flagella contain a “9 + 2” axoneme. Besides this core axoneme structure, other affiliated structures, including the fibrous sheath and outer dense fibers, are also assembled into the sperm flagella [37]. S-SOX5 was originally cloned from mouse testis [10] and it is able to activate transcription of a group of testis-related gene such as I*κ*B*β*, *ZNF230* and *Catsper1* [12, 13, 38] Given that S-SOX5 is only expressed in tissues with motile cilia, particularly in the post-meiotic round spermatids [11], we hypothesize that this transcription factor regulates a suite of genes for motile cilia formation/function, particularly for sperm flagella formation/function. Recent GWAS studies suggest that the *SOX5* locus is associated with male infertility [39], and the high expression level of S-SOX5 in the testis implies that S-SOX5 plays an important role in regulating expression of the genes that are essential for sperm function and male fertility.

The in vivo function of S-SOX5 is still not known. However, the unique exon not present in *L*-*SOX5* allows us to make mutant mice with disruption of S-SOX5 only. Using this model, we will be able to study the function of S-SOX5 in vivo, and probably identify the target genes regulated by S-SOX5 globally.

## Conclusions

This study demonstrates the molecular mechanism underlying the regulation of human *SPAG16L* by S-SOX5. S-SOX5 activates transcription of *SPAG16L* through specifically interacting with SOX5 binding sites at the *SPAG16L* promoter. The data suggest that S-SOX5 plays a regulatory role in the formation of cilia/flagella.

## Methods

### Luciferase reporter constructs

The *SPAG16L* promoter luciferase reporter fusion was constructed by cloning a 2 kb human *SPAG16L* proximal promoter region including the transcriptional start site and multiple putative SOX5 binding sites into the pGL3-basic vector (Cat.E1751, Promega). Similarly, another luciferase reporter fusion was made by cloning a shorter region of *SPAG16L* proximal promoter into the pGL3-basic vector. This transcriptional *luc* fusion construct contained the transcriptional start site of *SPAG16L* and adjacent two putative SOX5 binding sites. The primers for construction of the transcriptional *luc* fusions were listed in Table 1.

**Table 1:** Oligonucleotides used in this study

Primer name	Sequence (5′–3′)	Application
SPAG16L-promoterF1	GGTACCGGTCAAAGCGAAAGAAAACC	Forward primer for transcription fusion to *luc*
SPAG16L-promoterR2	CTCGAGGAACAGCGAAGACGCTACCC	Reverse primer for transcription fusion to *luc*
Human S-SOX5 forward	GTGCCATAGGAGCTGTGCATG	S-SOX5 expression
Human S-SOX5 reverse	GTTGGTCCTTCATTTGCCGAGC	
SOX5 RNAi (225) sense	AAAAATGATGCTGTCACCAAGGCAA	SOX5 RNAi construction
SOX5 RNAi (225) anti-sense	AAAGTTGCCTTGGTGACAGCATCAT	
SOX5 RNAi (1109) sense	AAAAGATTATGGGAGTGACAGTGAA	SOX5 RNAi construction
SOX5 RNAi (1109) anti-sense	AAAGTTCACTGTCACTCCCATAATC	
SPAG16L RT-PCRF	TTCAGACTGCTGCTTCCATC	Real-time PCR analysis of SPAG16L
SPAG16L RT-PCRR	TCGCCTGTACATAGATCCCA	
GAPDH RT-PCRF	GGAGGTGAAGGTCGGAGTC	Real-time PCR analysis of GAPDH
GAPDH RT-PCRR	GAAGATG GTGATGGGATTTC	
SPAG16L mutation 1F	TGCAATGCAAGCCAACCACCTACTGTATCTTGTCC	Mutation of SOX5 binding site (P-I site) at SPAG16L promoter
SPAG16L mutation 1R	GGACAAGATACAGTAGGTGGTTGGCTTGCATTGCA	
SPAG16L mutation 2F	GTTAACTAGGCAACACTACCGCCACGGTAACTGGG	Mutation of SOX5 binding site (P-II site) at SPAG16L promoter
SPAG16L mutation 2R	CCCAGTTACCGTGGCGGTAGTGTTGCCTAGTTAAC	
SPAG16L ChIP-aF	CTTGGGTGACTTCCAATTTTG	ChIP assays for SPAG16L site a
SPAG16L ChIP-aR	GTATCATTAACTACACTCCTC	
SPAG16L ChIP-bF	CCTACTTGAGGAGGAGAGTGGGA	ChIP assays for SPAG16L site b
SPAG16L ChIP-bR	TATCGCGTATGTATCAGAAGC	
SPAG16L ChIP-cF	GGAAGATCCTCTCAGCAATAAGAC	ChIP assays for SPAG16L site c
SPAG16L ChIP-cR	GAAGAACTATGGTGTTCAGC	

### Expression constructs or adenovirus expressing S-SOX5 and SOX5 RNAi constructs

*S*-*SOX5* expression plasmids, the adenovirus expressing *S*-*SOX5,* and the RNAi constructs targeting *SOX5* transcripts were generated previously in the laboratory [9]. Oligonucleotides used for generation of these constructs are listed in Table 1.

### Site-directed mutagenesis of SOX5 binding sites in the *SPAG16L* promoter

Two SOX5 binding sites in the *SPAG16L* promoter construct were mutated using a QuikChange II XL Site-Directed Mutagenesis Kit (Agilent Technologies) following the manufacturer’s instructions. Mutations at the *SPAG16L* promoter were verified by DNA sequencing. The mutagenic primers for construction of mutated SOX5 binding sites are shown in Table 1.

### Western blot analysis

Equal amount of proteins (50 µg/lane) were heated to 95 °C in sample buffer for 10 min, resolved in 10% SDS–polyacrylamide gels and then electro-transferred to polyvinylidene difluoride membranes (Millipore). After blocking in TBS-T buffer (Tris-buffered saline solution containing 5% non-fat dry milk and 0.05% Tween 20) for 1 h, the membranes were incubated with antibodies against SOX5 (Aviva Systems Biology, Santa Cruz, CA) or rabbit β-actin (Cell Signaling Technology, Danvers, MA) overnight at 4 °C. After being washed in TBS-T, the membranes were incubated with an anti-rabbit immunoglobulin conjugated with horseradish peroxidase (1:2000 dilution) at room temperature for 1 h. SOX5 or β-actin proteins were detected with SuperSignal West Pico Chemiluminescent Substrate (Pierce).

### Chromatin immunoprecipitation (ChIP)

ChIP assays were conducted using a ChIP assay kit (Millipore) according to the manufacturer’s instructions. Briefly, BEAS-2B cells were (CRL-9609) purchased from the American Type Culture Collection and infected with AdS-SOX5 for 48 h. After infection, the protein-DNA complexes from the cells were cross-linked by addition of 1% formaldehyde. The cells were suspended in SDS lysis buffer and were sonicated to shear DNA to 200–1000 bp fragments. Samples were precleared with protein A agarose/salmon sperm DNA (50% slurry) and were immunoprecipitated with antibodies against SOX5 or IgG. After washing the immunocomplexes with appropriate buffers, DNA was recovered by reverse cross-linking and purified by phenol/chloroform extraction followed by ethanol precipitation. The DNA fragments were used as a template for PCR reaction with primer sets (Table 1) flanking the SOX5 binding sites.

### Transient transfection and luciferase assays

Human bronchial epithelial BEAS-2B cells were cultured in BEBM and were plated 24 h before transfection. The cells were transfected with plasmids containing the wild-type or mutated *SPAG16* promoter using FuGENE6 transfection reagent (Roche). Co-transfection was performed with either empty vectors or S-SOX5 expression vectors (S-SOX5/pcDNA3). The cells were cultured for 48 h and the promoter activity was measured by the Dual-Luciferase Reporter Assay System (Promega). Luciferase activity was normalized to *Renilla* luciferase activity (control vector).

### Real-time PCR

Total RNA was extracted from BEAS-2B cells infected with indicated plasmids using TRIzol Reagent (Invitrogen) and was reversed transcribed to cDNA. The cDNA was used for PCR amplification of *SPAG16L, SOX5* and *GAPDH* with primers listed in Table 1. Real-time PCR was performed using 2× SYBR Green master mix (Bio-Rad).

## Abbreviations

SPAG16: sperm-associated antigen 16
ChIP: chromatin immunoprecipitation
